# Endogenous phytohormones of frankincense producing *Boswellia sacra* tree populations

**DOI:** 10.1371/journal.pone.0207910

**Published:** 2018-12-19

**Authors:** Abdul Latif Khan, Fazal Mabood, Fazal Akber, Amjad Ali, Raheem Shahzad, Ahmed Al-Harrasi, Ahmed Al-Rawahi, Zabta Khan Shinwari, In-Jung Lee

**Affiliations:** 1 Natural & Medical Sciences Research Center, University of Nizwa, Nizwa, Oman; 2 Department of Biological Sciences & Chemistry, University of Nizwa, Nizwa, Oman; 3 School of Applied Biosciences, Kyungpook National University, Daegu, South Korea; 4 Department of Biotechnology, Quaid-e-Azam University, Islamabad, Pakistan; Fred Hutchinson Cancer Research Center, UNITED STATES

## Abstract

*Boswellia sacra*, an endemic tree to Oman, is exposed to man-made incisions for commercial level frankincense production, whereas unsustainable harvesting may lead to population decline. In this case, assessment of endogenous phytohormones (gibberellic acid (GA), indole-acetic acid (IAA), salicylic acid (SA) and kinetin) can help to understand population health and growth dynamics. Hence, it was aimed to devise a robust method using Near-Infrared spectroscopy (NIRS) coupled with multivariate methods for phytohormone analysis of thirteen different populations of *B*. *sacra*. NIRS data was recorded in absorption mode (10000–4000 cm^-1^) to build partial least squares regression model (calibration set 70%). Model was externally cross validated (30%) as a test set to check their prediction ability before the application to quantify the unknown amount of phytohormones in thirteen different populations of *B*. *sacra*. The results showed that phytohormonal contents varied significantly, showing a trend of SA>GA/IAA>kinetin across different populations. SA and GA contents were significantly higher in Pop13 (Hasik), followed by Pop2 (Dowkah)–an extreme end of *B*. *sacra* tree cover in Dhofar region. A similar trend in the concentration of phytohormones was found when the samples from 13 populations were subjected to advance liquid chromatography mass spectrophotometer and gas chromatograph with selected ion monitor analysis. The current analysis provides alternative tool to assess plant health, which could be important to in situ propagation of tree population as well as monitoring tree population growth dynamics.

## Introduction

The genus *Boswellia* (Burseraceae) comprises about twenty species widely distributed in Arabia (South Yemen, Oman), the northeastern coast of Africa (Ethiopia, Somalia, Sudan) and north-western India [1]. The ole-gum resin (frankincense) of *Boswellia* trees is reported to be the most important commercial and phytopharmaceutical source [2]. *Boswellia sacra*, endemic to Dhofar region of Oman [3], is one of the economically important frankincense- *or* olibanum*-*producing trees of the Sultanate of Oman where it is known as "meqerot" and its oleo-gum resin is called Frankincense (Luban) [4,5]. The gum resin of *Boswellia sacra* Flueck has many cultural, economic and medicinal values [2] and is used as analgesic, hepatoprotective, antioxidant, anticoagulant, tumor suppressive, antinflammatory, and cardioprotective, and in the treatment of gastric, hepatic, and skin disorders [6].

In addition to the importance of *B*. *sacra* tree, very less has been known about its physiology and ecology. The wounding/tapping practice of the tree is carried out at different part of the stem with an axe to obtain frankincense. A tree is normally given 8–10 incisions within the six months of the dry season. A wound to the stem is about 5cm^2^ and a depth of about 5mm [3,7]^.^ After a week of incisions, the crystalline resin is harvested and thus followed by another round of incisions. However, the number of tapping points is dependent on tree diameter. It has been found that with the increase in frankincense demand, the trapping points may increase in individual tree, impacting tree growth and physiology [3,8,9]. Contrarily, the carrying capacity of the tree is less than nine tapping points. Frankincense yield per tree per season varies up to 41gm depending on the size/age of the tree and seasonal conditions [10]. The production of Frankincense is high during the initial round of incisions, whereas, this gradually reduces with the number of preceding incisions [3,10–15] thus effecting the overall tree population and their capacity to produce.

The tree responds to incisions by activating its defense mechanism through the production of chemical messengers such as phytohormones to reduce the negative impacts of resin production [16]. Phytohormones, such as gibberellic acid, indole acetic acid, kinetin, and salicylic acid etc have been known to confer abiotic (heat, drought, heavy metal, salinity etc) and biotic (herbivory and pathogenic infections) stresses [17–20]. In addition, the role and function of these endogenous phytohormones varies greatly among the plants belonging to same species but expose to different climatic conditions[21]. Recent studies have targeted responses of endogenous abscisic acid, (ABA), gibberellic acid (GA), indole acetic acid (IAA) and salicylic acid (SA) drought stress in *Pinus* trees cultivated sites [22,23]. However, since the climate, growth condition and the kind of plant varies, it is evident that the resulting phytohormonal responses to environmental stimuli will be different [20]. This has been rarely explained across various populations of wildly growing plants and their populations. Studies describing the physiological dynamics were also helpful to predict the health of tree population and regenerating capacities.

There are several analytical methods available to understand the dynamics of endogenous phytohormones using spectroscopic (gas chromatography and mass spectroscopy with selected ion monitor–GC-MS/SIM; ultra-high performance liquid chromatography coupled with MS–UPLC/MS and nuclear magnetic resonance–NMR) and molecular (polymerase chain reaction–PCR and real-time PCR) [24–28]. In addition, utilizing Near Infrared spectroscopy (NIRS) as a vibrational spectroscopy technique in combination with multivariate regression analysis can help understanding the variation among different samples as well as quantifying some of the essential metabolites in plants [29–31]. NIRS can measure the chemical texture and composition of samples based on the absorption of the near infrared radiation by bonding between C–H, O–H and N–H and this results in overtones and combination of bands or peaks detectable in a wavelength ranging from 780–2500 nm [29–34]. A few reports have documented the utility of NIRS to classify the biological samples such as seed lots of agricultural crops [35], gourd seeds [36], spinach seeds [37] and soybean seeds [38]. Furthermore, the NIRS has also helped in separating viable and non-viable tree seeds [39]. Moreover, recent studies have shown the use of NIRS models in young and mature leaf identification of Amazonian tree species [40]. In addition, coupling NIR with statistical modeling such as partial least squares regression contributed in understanding the growth and dynamics of specific ecosystem [41–44]. The NIRS analysis are robust tool, however, these have not been applied to tree populations. Throughout literature, we could not find any quick procedure involving NIRS spectra based PLS regression model to quantify endogenous phytohormones in tree population, hence we constructed it. To further validate the models, variation in phytohormones of different tree population was also assessed. On the other hand, studies on *Bo*s*wellia* sacra and its population are scarce except for *Boswellia papyrifera* [9,45]. The current study aimed to use NIR-based methods supplemented with PLS regression modeling to quantify the endogenous phytohormones (gibberellic acid, indole acetic acid, kinetin, and salicylic acid) and their variations among 13 different populations of *B*. *sacra*. The NIR based quantification of phytohormones were also validated by advanced chromatographic and spectrophotometric techniques.

## Materials and methods

### Sample collection and identification

**Fig 1:**
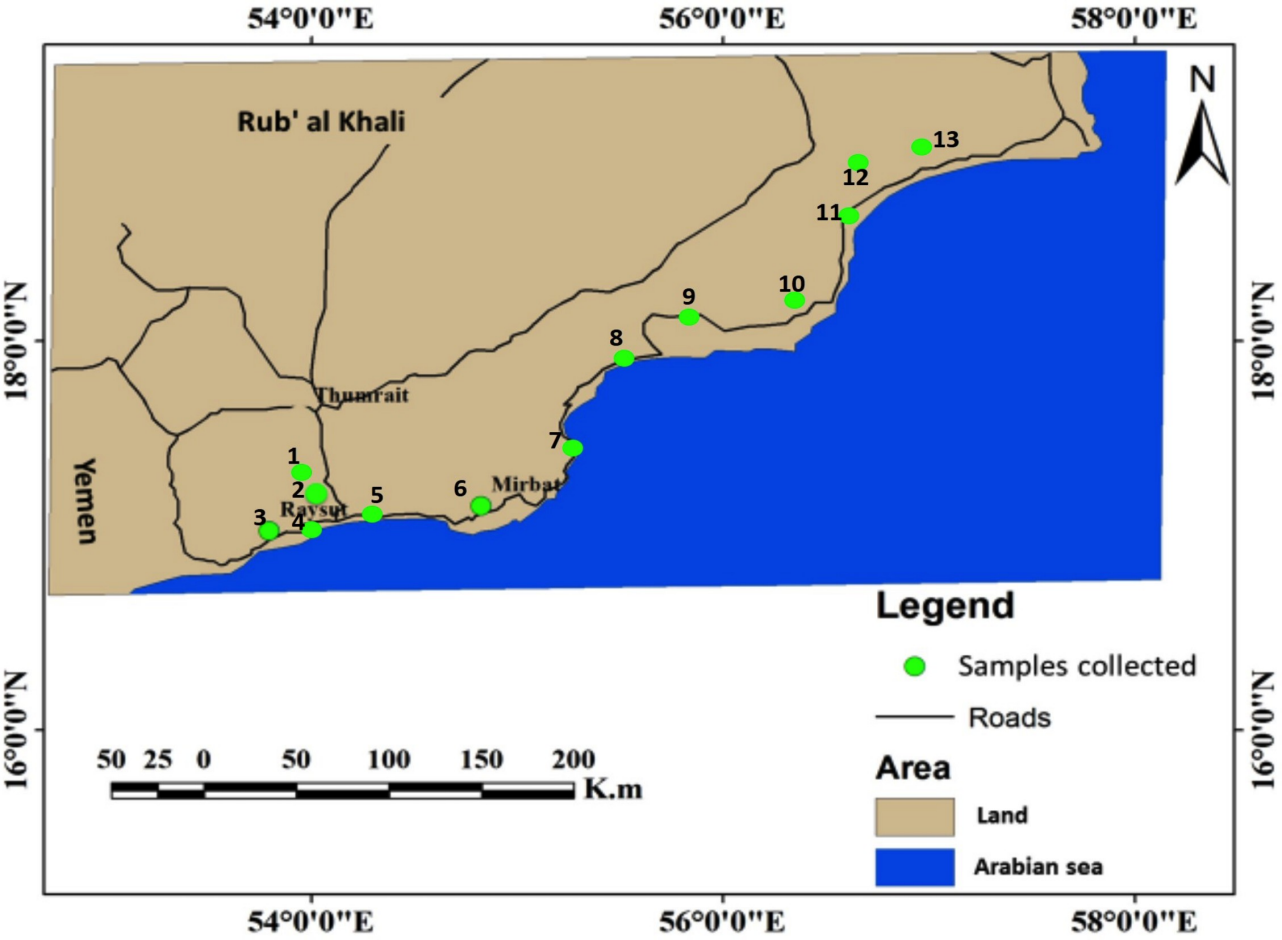
Sampling collection area. Map of the Sultanate of Oman showing the sample collected from the hot-spot areas of *B*. *sacra* population. All the rhizospheric samples were collected from three different locations, with same climatic conditions. The map for sample collection was made in ArcGIS v9.3.1 (Redlands, CA, USA; http://www.esri.com/software/arcgis/eval-help/arcgis-931).

We collected samples from the trees that grew wildly in the area, where no specific permit was required for taking samples. Our study did not use any endangered or protected species. The trees used for sampling were treated ethically, and our study did not harm the local environment. Leaf samples were collected from the 13 different locations of the wildly growing *B*. *sacra* populations at Dhofar region, Sultanate of Oman (Fig 1). These 13 plant collection sites are the key populations of the *B*. *sacra* tree in Dhofar region. From each population, 50 trees (comprising of 10 trees per replica; with a total 5 replicates per population) of uniform height, width and age were selected. Whereas, each population was ~5 to 50 km distance a part from each other (Fig 1; S1 Table). The mature leaf samples of uniform size were preserved in liquid nitrogen and transported to the lab. The fresh leaf parts (1.0g) were extracted with methanol (MeOH) for 24hrs at 4°C in five different flasks. The MeOH extract was centrifuged at 4°C (13,500 rpm; 20 minute). The pH of the clear supernatant was adjusted to 3.8 as per protocol of Lee et al. [46] and Schmelz et al. [47].

### Standards preparation

In this study four different plant hormones standard solutions i.e. IAA, GA, SA & Kinetin in the range of 0.1μM to 250 μM concentration were prepared and were used for making PLSR models and for their cross validation. The total number of standards solution used was 260 i.e. 65 of each individual hormone. The standard solution of each individual phytohormone was split into two sets, a training set (70% of the standard solutions) and a test set for external validation (30% of the standard solutions). The training set was used for building the PLS regression model, while the test set was used to check the prediction ability of the regression model as an external validation set. Samples are estimated set by using optimized PLSR model. This was followed with the comparative assessment of test with 13 samples of populations as unknown [48–49].

### NIR spectral measurement

All phytohormonal standards (AppliChem GmbH, Darnstadt, Germany) from 0.1μM to 250 μM and different fractions of *Boswellia sacra* populations were prepared in methanol and were measured by Frontier IR/NIR system model number (L1280034) by PerkinElmer in the wavelength range from 10000 to 4000 cm^-1^, in the absorption mode using a 0.2 mm path length CaF_2_ sealed cell at 2 cm^-1^ resolution [49].

### PLS regression model and analysis of data

Multiplicative signal correction (MSC) and 1^st^ derivatives spectral pre-treatments were used to optimize the prediction ability of PLS regression models. Root mean squared error of prediction (RMSEP), root mean square error of cross validation (RMSEC), coefficient of determination (R^2^), and the number of factors were used as a tool of measures of better performances after the application of spectral pre-processing on PLS regression (PLSR) models. The PLS regression models will be the best having small values of RMSEC, RMSEP, less number of factors but with the value of R^2^ high. The results are given in Table 1.

**Table 1:** Selection of pre-processing for NIRS analysis of phytohormones across different populations of *B*. *sacra*.

Type of spectra	Pre-processing	PLS		PLS		# of factors
		**RMSEC**	**R**^**2**^	**RMSEP**	**R**^**2**^	
**Indole Acetic Acid (IAA)**						
Full Spectra (4000 to 10000 cm^-1^)	Without pre-processing	6.23	0.829	7.58	0.746	5
Spectra (4000 to 6000 cm^-1^)	Without pre-processing	6.94	0.7877	12.8	0.276	5
Full Spectra (4000 to 10000 cm^-1^)	MSC	4.27	0.91	5.84	0.85	7
Spectra (4000 to 6000 cm^-1^)	MSC	6.54	0.80	8.59	0.67	5
Full Spectra (4000 to 10000 cm^-1^)	1^st^ derv. with 13 smoothing points	0.470	0.99	2.97	0.94	7
**Spectra (4000 to 5000 cm**^**-1**^**)**	**1**^**st**^ **derv. with 13 smoothing points**	**5.10**	**0.86**	**9.69**	**0.58**	**4**
**Gibberellic acid (GA)**						
Full Spectra (4000 to 10000 cm^-1^)	Without pre-processing	4.46	0.91	8.20	0.70	7
Spectra (4000 to 7000 cm^-1^)	Without pre-processing	5.300	0.877	12.8	0.34	7
Full Spectra (4000 to 10000 cm^-1^)	MSC	4.31	0.91	4.51	0.91	7
Spectra (4000 to 7000 cm^-1^)	MSC	8.31	0.69	7.37	0.76	5
Full Spectra (4000 to 10000 cm^-1^)	1^st^ derv. with 13 smoothing points	0.903	0.99	1.29	0.98	7
**Spectra (4000 to 7000 cm**^**-1**^**)**	**1**^**st**^ **derv. with 13 smoothing points**	**4.31**	**0.89**	**6.85**	**0.79**	**7**
**Salicylic acid (SA)**						
Full Spectra (4000 to 10000 cm^-1^)	Without pre-processing	0.08	0.99	1.36	0.92	7
Spectra (4000 to 6000 cm^-1^)	Without pre-processing	5.98	0.10	5.89	0.1291	1
Full Spectra (4000 to 10000 cm^-1^)	MSC	0.9697	0.97	0.9880	-6.94	5
Spectra (4000 to 6000 cm^-1^)	MSC	1.78	0.65	12.16	-8.36	3
Full Spectra (4000 to 10000 cm^-1^)	1^st^ derv. with 13 smoothing points	0.85	0.92	12.48	-8.85	4
**Spectra (4000 to 6000 cm**^**-1**^**)**	**1**^**st**^ **derv. with 13 smoothing points**	**1.61**	**0.71**	**13.02**	**-9.13**	**4**
**Kinetin**						
Full Spectra (4000 to 10000 cm^-1^)	Without pre-processing	0.715	0.99	1.45	0.92	7
Spectra (4000 to 6000 cm^-1^)	Without pre-processing	3.71	0.93	11.66	0.4018	1
Full Spectra (4000 to 10000 cm^-1^)	MSC	5.94	0.84	8.59	0.67	4
Spectra (4000 to 6000 cm^-1^)	MSC	4.37	0.91	14.52	0.07	4
Full Spectra (4000 to 10000 cm^-1^)	1^st^ derv. with 13 smoothing points	2.63	0.96	7.93	0.72	5
**Spectra (4000 to 7000 cm**^**-1**^**)**	**1**^**st**^ **derv. with 13 smoothing points**	**5.99**	**0.84**	**9.63**	**0.59**	**3**

Partial least square (PLS); Multiplicative signal correction (MSC); RMSEC (root mean square error of cross validation); RMSEP (Root mean square error of prediction

**Fig 2:**
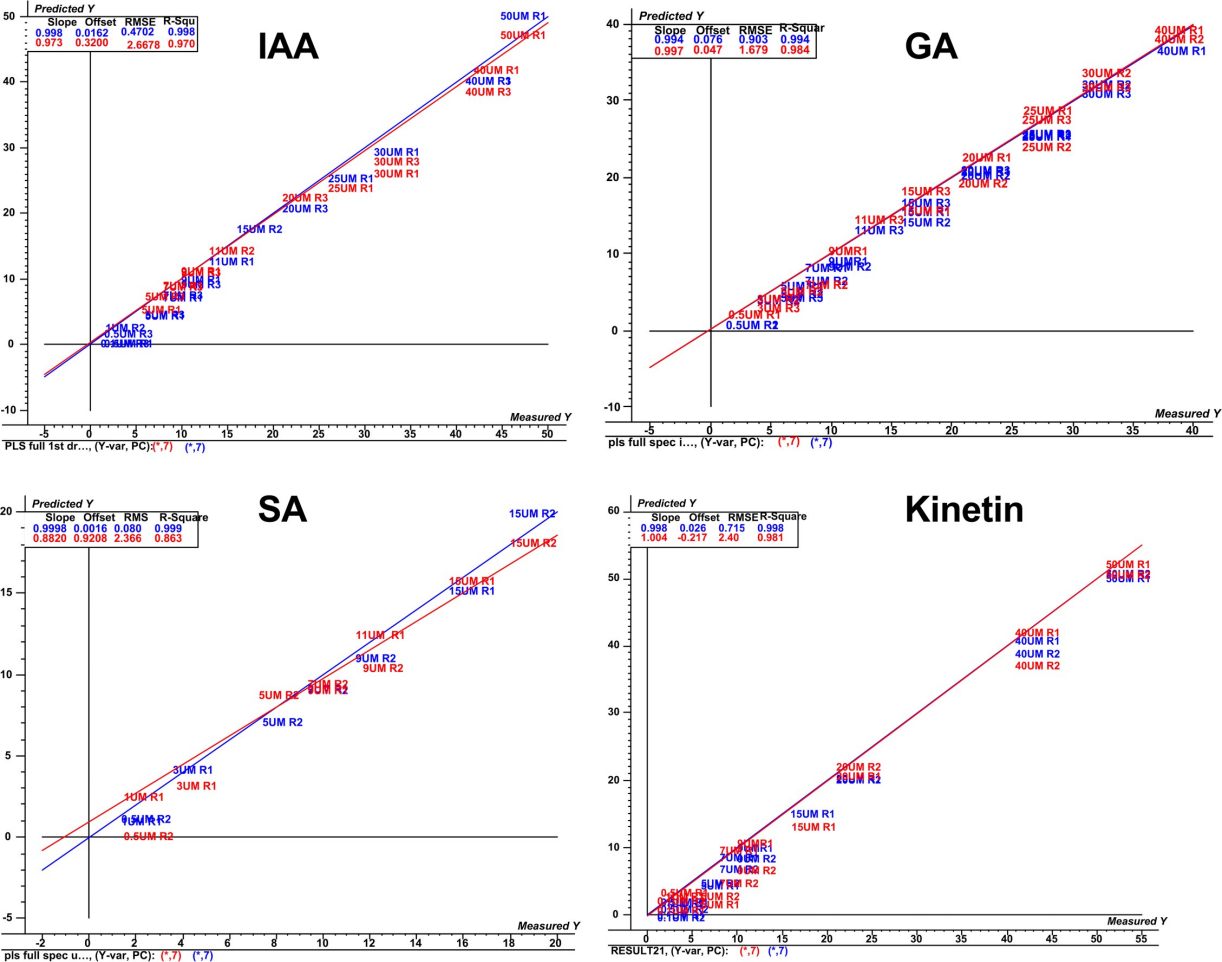
NIRS data based regression analysis. NIRS data spectra based PLS regression plot for IAA, SA, kinetin and GA with data inclusion for calibration set (70% standard solution).

PLS first extracts set of latent factors which usually explain the level of co-variance between the independent **“X”** and dependent “**Y”** variables^44^. In current study, the PLS finds a set of orthogonal components that maximize the level of explanation of both “**X”** and “**Y”** provides a predictive equation for “**Y”** in terms of the “**X**’s”. The regression step predicts values of the dependent-variables using the decomposition of the independent-variables as previously elucidated [42–44]. The PLS regression models of 13 *B*. *sacra* populations and relative phytohromonal variation are predicted here and shown in Fig 2. The results showed high level of correlationship between the “**X”** variables (spectra) and **Y (**the concentration) as shown in Table 2. The root mean squared error of cross-validation (RMSECV) value for PLS models are shown in Table 2 as an internal cross validation tool using random cross validation. PLS regression models having smaller values of RMSECV and the higher correlation with less number of factors are the indicative to have better prediction ability. RMSECV formula is given in Eq 1:

$$RMSECV=\sqrt{\frac{\sum_{i=1}^{n}{\left(y_{i}-{\hat{y}}_{i}\right)}^{2}}{n}}$$

**Fig 3:**
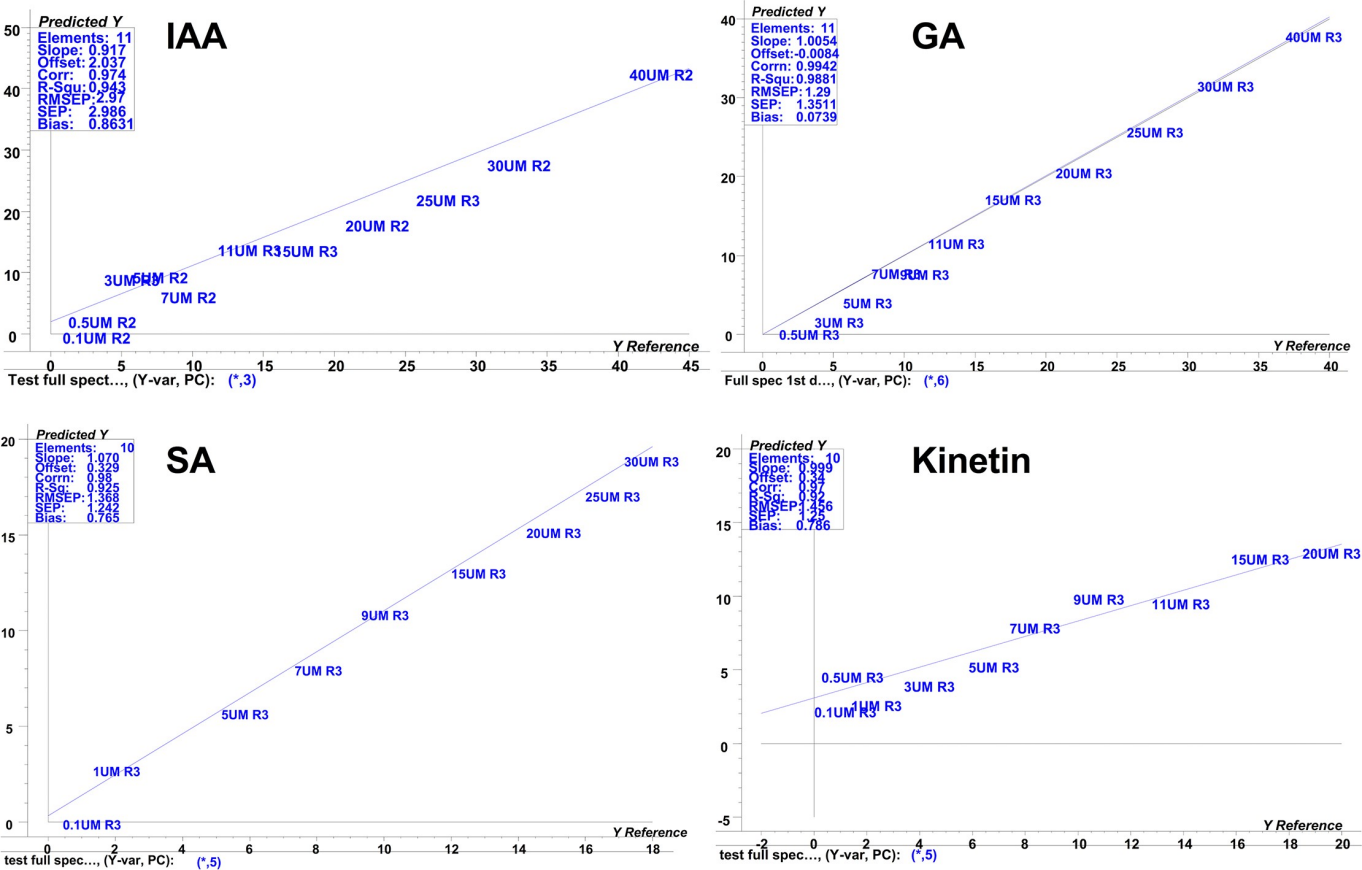
Prediction plot analysis. Prediction plot for the cross validation test (30% standard solution) of IAA, SA, Kinetin and GA as an external validation.

Where *y*_*i*_ is the actual value, ${\hat{y}}_{i}$ is the estimated value by the model, and *n* is the number of sample left out in the cross-validation procedure. Factor loading plot indicates how much of the variation of the spectral data contribute to building the PLS regression model. PLS regression models were then tested on the test set of all the four standards as an external cross validation set to check their prediction ability and performances in terms of RMSEP values (Fig 3).

**Table 2:** PLS regression model for the phytohomornal analysis from thirteen different populations of *B*. *sacra*.

Parameters	Indol acetic acid (IAA)	Giberellic acid (GA)	Salicylic acid (SA)	Kinetin
Parameters for PLS regression models				
RMSEC	0.47	0.90	0.08	0.715
R^2^	0.99	0.99	0.99	0.99
# of factors	7	7	7	7
Prediction performance Parameters of PLS regression models				
RMSEC	2.97	1.29	1.36	1.45
R^2^	0.94	0.98	0.92	0.92
# of factors	3	6	5	5

RMSEC (root mean square error of cross validation); RMSEP (Root mean square error of prediction

As can be seen from Table 2 the minimum value of RMSEP indicates very good prediction made by PLSR model using training set. RMSEP is calculated by formula in Eq 2:

$$RMSEP=\sqrt{\frac{\sum_{i=1}^{n_{t}}{\left(y_{t,i}-{\hat{y}}_{t,i}\right)}^{2}}{n_{t}}}$$

Where *y*_*t*,*i*_ is the actual value, ${\hat{y}}_{t,i}$ is the value predicted by PLSR model using test set. The minimum value of RMSEP leads and indicates less error for future plant samples unknown prediction. Microsoft Excel 2010 and the Unscrambler version 9.0 were used for statistical analysis as described by Al-Harrasi et al [48]. Multivariate calibration technique such as PLS regression was used to construct a mathematical model that relates the multivariate response (spectrum **X**) to the concentration of different phytohormones i.e. (**Y**), and such a model was used to efficiently predict the concentrations of phytohormones in different populations of *B*. *sacra*. Spectral pretreatments such as MSC, 1^st^ derivative function with Savitzky-Golay smoothing points were applied. External cross validation was used to validate the PLSR models built with the test set. As an internal indicator measuring tool Root Mean Square Error of Cross-Validation (RMSECV) was used to check the performance of the PLS models, while Root Mean Square Error of Prediction (RMSEP) as a cross validation external tool.

### Quantification of phytohormones by advanced chromatographic and spectrophotometry

Salicylic acid (SA) was extracted from lyophilized tissues (200 mg) and quantified as described by Seskar et al [50]. Briefly, the powdered tissues were extracted using methanol (90 and 100%) and centrifuged at 5000×g and 4°C, after which the dry pellets were re-dissolved in trichloroacetic acid (5%). The resulting supernatants were separated using a 99:99:1 (v:v:v) mixture of ethyl acetate, cyclopentane, and isopropanol, and the organic layer was dried using nitrogen stream and re-suspended in methanol (70%). The semi-pure extract was then analyzed using high-performance liquid chromatography coupled with a Shimadzu fluorescence detector, C18 reverse-phase column (S1 Table), and flow rate of 1.0 ml/min. The SA analyses were repeated three times.

Endogenous GAs were extracted from freeze-dried plant samples (0.5 g) and quantified as described by Lee et al. [46]. Before partitioning, deuterated ([17, 17-_2_H_2_] GA_4_; 20 ng each) internal standards. The upper organic portions were dried using a rotatory evaporator, and 60% methanol (MeOH) was added, while maintaining the pH at 8.0±0.3. Following the detailed steps of chromatography and extraction, the different GA fractions of the semi-pure samples were separating using HPLC, and the resulting pure fractions were identified and quantified using GC-MS/SIM (S2 Table). Full-scan mode (the first trial), three major ions of the supplemented internal standards ([17-^2^H^2^] GAs), and the plant’s GAs were monitored simultaneously, and the same was done for the endogenous GAs in different populations of the tree. The endogenous GAs was quantified by comparing the peak area ratios of sample GAs to those of corresponding internal standards. The retention time was determined using hydrocarbon standards to calculate the Kovats retention index (KRI), and GA quantification was based on the peak area ratios of non-deuterated (extracted) GAs to deuterated GAs (S2 Table). The experiment was repeated three times.

Plant IAA and kinetin quantifications were performed according to the method of Park et al. [51] and Pan et al. [52] using advanced Liquid Chromatography Electrospray-Ionization Quadrupole Time-of-flight (LC-ESI-QTOF) Tandem Mass Spectrometry. In brief, a 0.5g of freeze-dried leaf sample from different populations were ground finely and added it with 2.0ml buffer [2-propanol: H_2_O: concentrated HCl (2: 1: 0.002, vol / vol / vol)]. The extraction solution was incubated at 4°C on shaker for 30 min. It was then added with 2.0ml of dichloromethane to each sample and after incubation, it was centrifuged at 13,000g for 5 min at 4°C. The lower layer was transferred to another tube and concentrated using nitrogen stream. The resulting semi-pure mixture was re-dissolved in 10 μl methanol. The samples were analyzed in a XEVO G2-S QTOF Spectrometer (Waters, USA) connected with Ultra Performance Liquid Chromatography (UPLC, Waters, USA) using Acquity UPLC BEH C18 1.7 μM (2.1 x 100 mm) column. All the experiments were repeated at least three times.

### Statistical analysis

In addition, to assess the different population structure and its effect on the regulation of phytohormones, we used analysis of variance (ANOVA) with multiple comparisons among populations using Tukey’s test (*P<0*.*05*). The data was statistically analyzed for standard deviation, mean values, and graphical presentation using GraphPad Prism v6.01 (GraphPad Software Inc., San Diego, CA, USA). An additional analysis of non-metric multidimensional scaling (nMDS) was performed to understand ordination based on a distance or dissimilarity matrix [44].

## Results

### NIR spectral measurement of *B*. *sacra* populations

**Fig 4:**
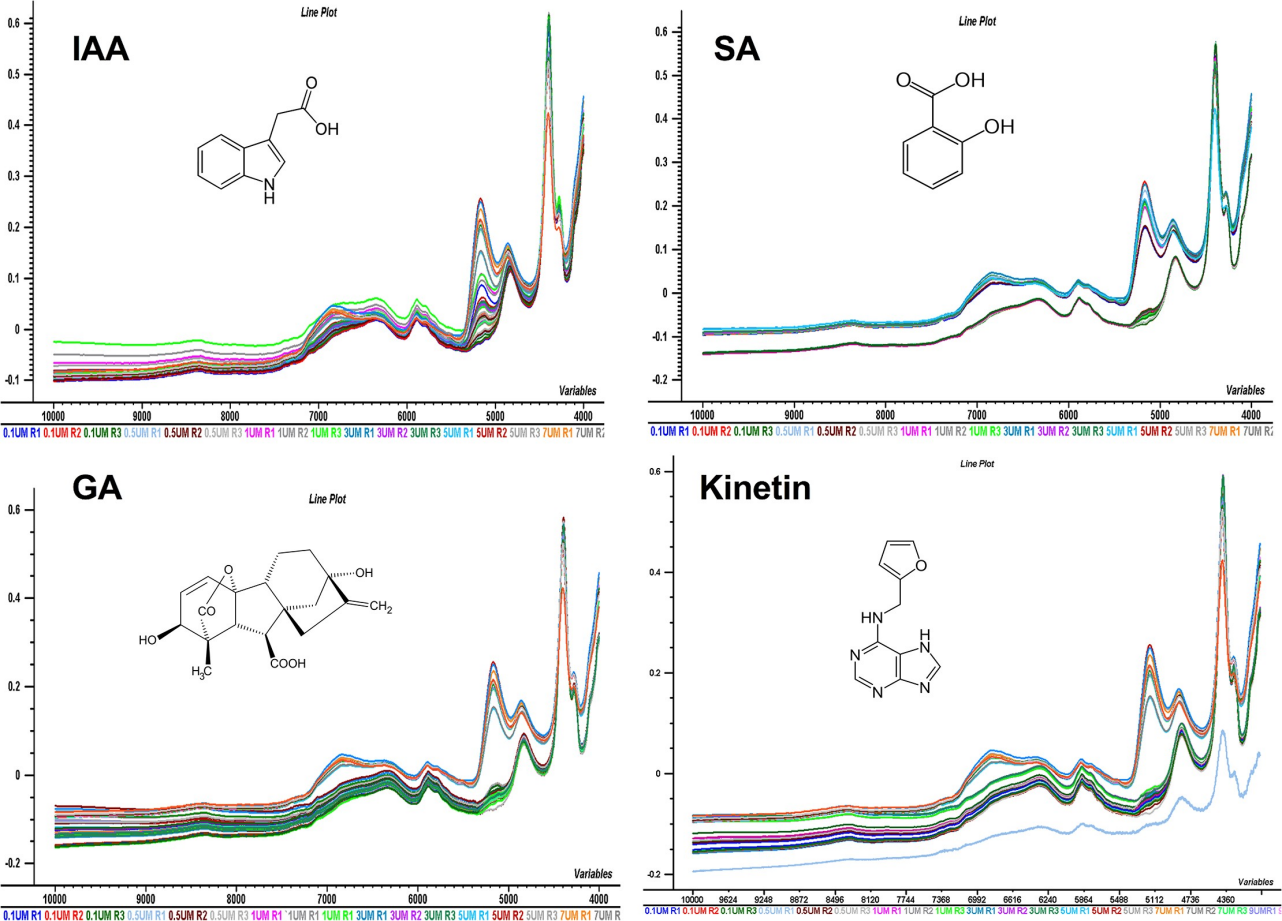
NIRS data spectra of populations. NIRS data spectra for IAA, SA, Kinetin and GA and 13 different populations of *B*. *sacra* samples. The figure elucidates the scattering effect due to absorbance without preprocessing on wavelength range from 10000 to 4000 cm^-1^.

**Fig 5:**
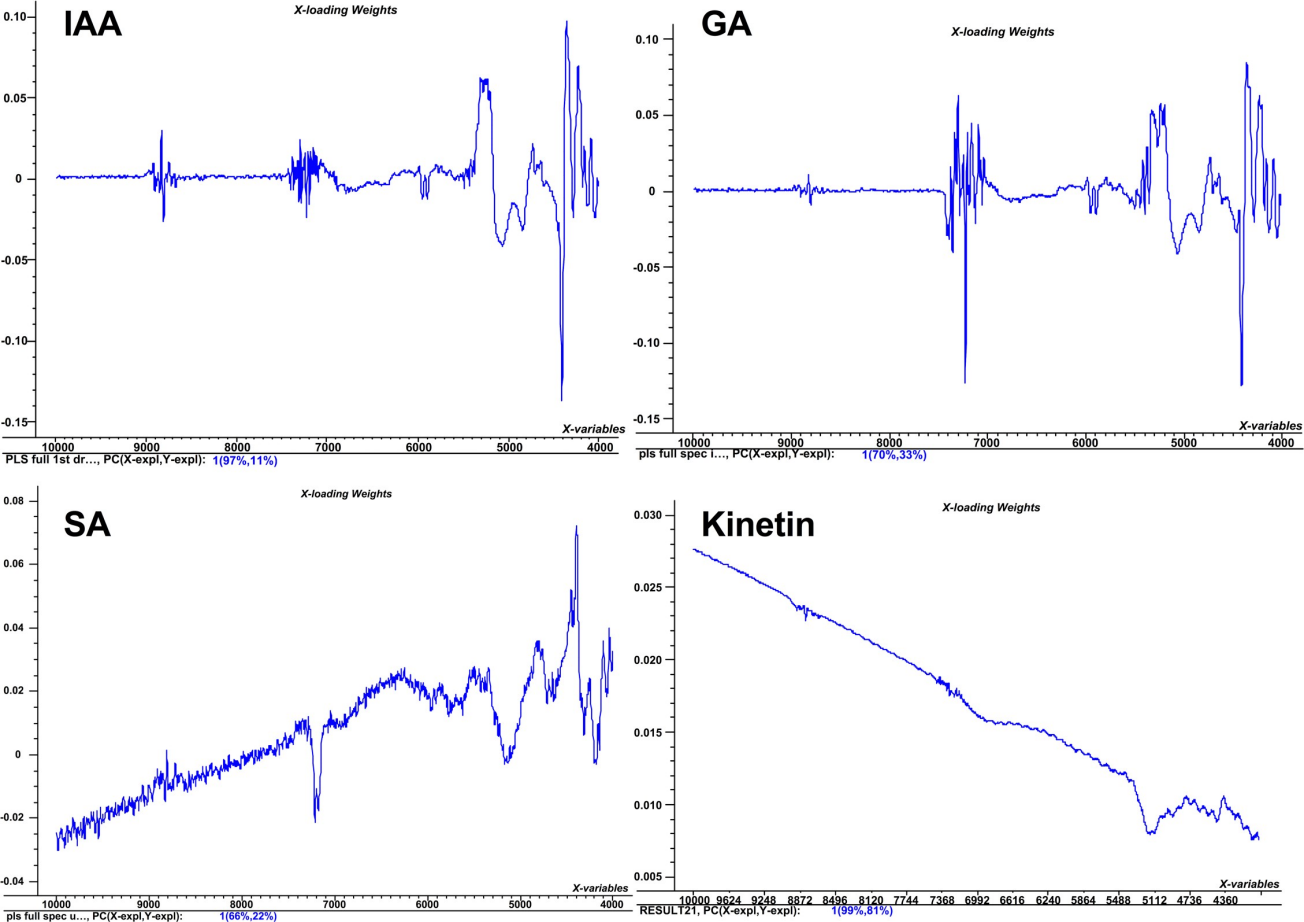
Factor loading plots for standards. The factor loading plot for PLS regression model for IAA, SA, Kinetin and GA.

The samples from 13 populations of *B*. *sacra* tree and the different concentrations of phytohromones i.e. IAA, GA, SA, and Kinetin were subjected to NIR spectroscopy for the measurement of their absorption in the range of wavelength from 10000 to 4000 cm^-1^. The resulting spectra are presented in Fig 4. It can be seen from the spectral data of 13 populations and the individual phytohormonal oscilation that there are scattering effects due to un-smooth reflection. NIR spectrum of gibberellic acid depicted the regular pattern of absorption with absorption peaks in the range of 4500–5200 cm^-1^ and 7100–73000 cm^-1^. The FT-NIR spectrum of gibberellic acid was evaluated where peak observed at 4500 cm^-1^ represents C-H combination band. The absorption band centered at 4700 cm^-1^ is due to R-OH group. The absorption at 5200 cm^-1^ represents C = O stretching. Multiple peaks in the region 7000–7300 cm^-1^ represent C-H, CH2, CH3 (second overtones) and O-H first overtone, respectively as shown in Figs 2–5. The indole-acetic acid (IAA) exhibited the absorption band at 4000–4300 cm^-1^ corresponds to C-H, CH2 combination region. The absorption band at 4500 cm^-1^ represents N-H combination band while the band at 5200 cm^-1^ represents C = O stretching group. The peak at 5900 cm-1 represents 1st overtone of aromatic C-H group. The absorption band centered at 7100 cm^-1^ represents O-H 1st overtone region while the band around 9100 cm^-1^ correspond to 2nd overtone of aromatic C-H.

NIR spectrum of SA exhibited absorption bands at 4000–4300 cm^-1^ correspond to C-H, CH2 combination region. The absorption band at 5000 cm^-1^ represents O-H combination region. The peak centered at 7100 cm^-1^ corresponds to represents O-H 1st overtone region while 5200 cm^-1^ represents C = O stretching group. The band at 7100 cm^-1^ represents 1st overtone of aromatic O-H group. The NIR spectrum of kinetin exhibited unique spectral features. The absorption band observed at 4300–4500 cm^-1^ corresponds to N-H combination peaks. The absorption band centered at 5100 cm^-1^ corresponds to C-N combination peaks. The absorption band at 6700 cm^-1^ is due to N-H 1st overtone while band at 8800 cm^-1^ represents 2nd overtone of C-H, as shown in Figs 4 and 5.

**Fig 6:**
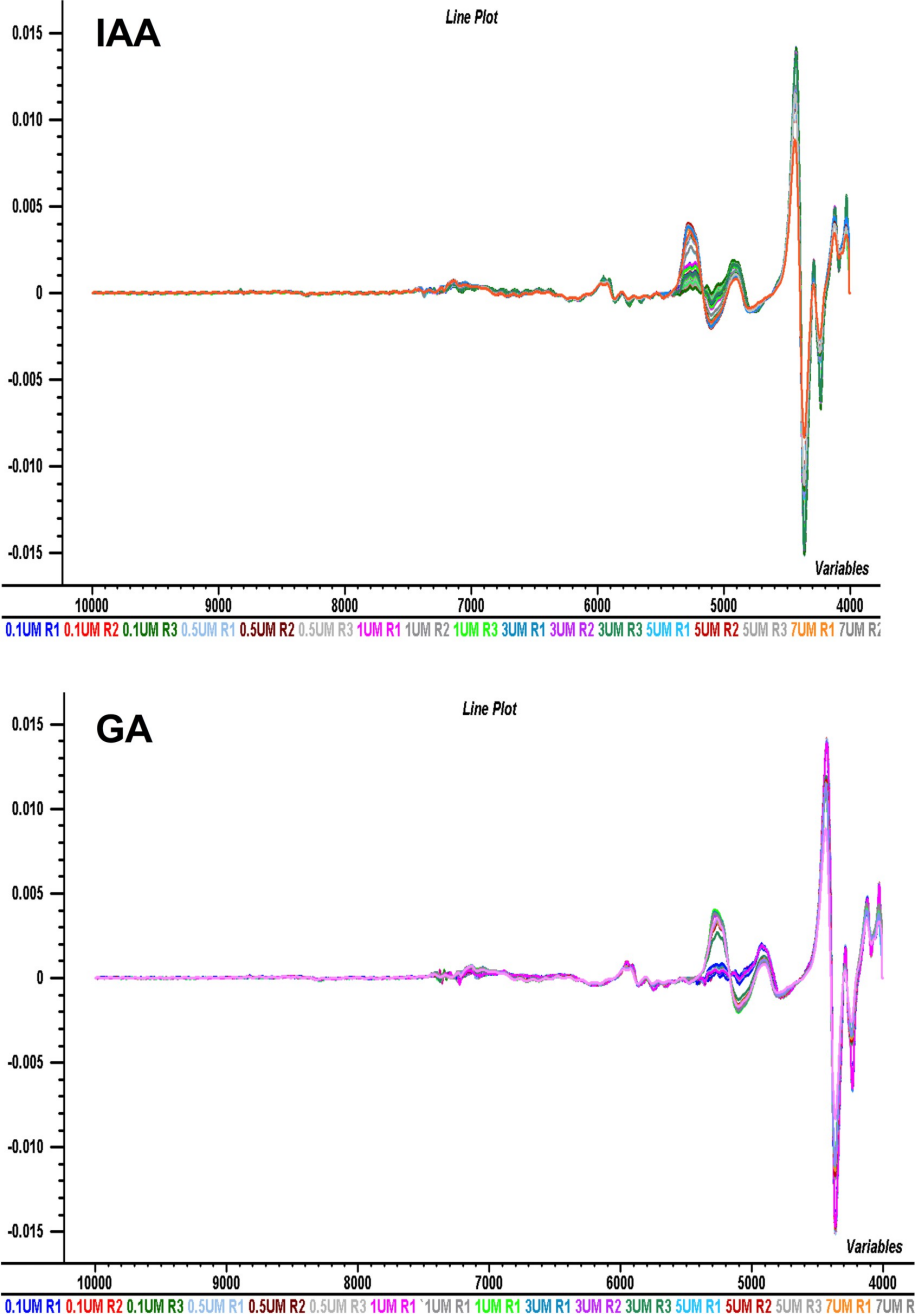
NIRS data spectra for samples. NIRS data spectra for IAA and GA and 13 different populations of *Boswellia sacra*. Samples are processed after 1^st^ derivative functions with Savitzky-Golay smoothing.

As it can be seen from Table 1, that in case of IAA and GA, the use of 1^st^ derivative functions with Savitzky-Golay 13 points smoothing for full wavelength spectra has improved the prediction ability of PLS regression model in case of IAA and GA. All of them have minimum error and the highest correlation. The PLS models with 1^st^ derivative functions with Savitzky-Golay smoothing 13 points for wavelength range from 4000–6500 cm^-1^ were used to predict the amount of IAA and GA in different *Boswellia* samples. Fig 6 shows the changes in NIR spectra for IAA and GA standards as well as for *Boswellia* sacra samples after the application of 1^st^ derivative functions pre-processing treatment. The prominent absorption peaks can be seen from the spectra in Fig 6 are in between the wavelength range from 4000 cm^-1^ to 6500 cm^-1^ for all the samples. While in case salicylic acid and kinetin the full wavelength spectra without any pre-processing afforded the optimum PLS model parameters. It means the spectral treatment has not improved the prediction ability of PLS regression models in case of SA and Kinetin.

### PLS regression of phytohormones

The amount of gibberellic acid, indole acetic acid, kinetin, and salicylic acid among 13 different populations of *Boswellia* sacra was assessed for PLS regression model. Using 70% of the standards values as a regression set for making the PLS regression models while keeping optimum spectral treatment conditions (Figs 2–6). It has been known through previous studies, that the increasing number of predictor (s) also increase the number of cases, and/or if the predictor variables are highly interrelated then the PLS will show the best predictive model of a given samples^42^. Similary the factor loading plots of PLS regression models for explained variation are shown in Fig 5. This shows the factor loading plot for factor 1 of the four optimum PLS regression models. In case of IAA the factor 1 loading plot contributes 97%, for GA 70%, for SA 66% while in case of kinetin it contributes 99%.

### Quantification of endogenous phytohormones in different populations

After external cross validation on a test set the PLS regression models were then used for prediction the amount of four plant hormone i.e. IAA, GA, SA & Kinetin in different populations of *B*.*sacra* tree. The results showed that the phytohormonal contents varied significantly (p<0.001) across different populations of the *B*. *sacra* tree (Table 3). Among phytohormones, SA content was highly significant (p<0.0001) in its content as compared to other phytohormones. Kinetin, on the other hand was significantly (p<0.0001) lower than IAA, GA and SA contents. GA and IAA showed similar moderate concentrations. Thus, the overall trend in phytohormonal content is SA>IAA/GA>Kinetin (Table 3). Individually, IAA content was significantly higher in Pop13 (Hasik area), an extreme end of *B*. *sacra* tree cover in Dhofar region. It was nearly two-fold higher in Pop13 as compared to other populations (Table 3). This increase in IAA content was followed by Pop11 and Pop12, which were also collected from start and middle of Hasik area respectively. Interestingly, the IAA content was also higher in Pop2, showing an un-significant difference (p<0.084) in comparison with Pop11 and Pop12 populations. In case of the other populations, a moderate to lower IAA content was found (Table 3).

**Table 3:** The predicted amounts of endogenous phytohormones in different populations of *B*. *sacra* based on optimum PLS regression models.

Populations	IAA μg^-1^ g FW	GA μg^-1^ g FW	SA μg^-1^ g FW	Kinetin μg^-1^ g FW
POP1	123.7±2.31	112.4±0.21	136.9±0.43	61.5±1.34
POP2	143.6±1.01*	164.7±0.42*	193.3±0.41*	42.6±1.4
POP3	119.8±1.0	151.7±0.76	172.5±0.52	51.0±2.0
POP4	108.7±0.81	143.1±0.56	152.8±0.76	53.8±0.98
POP5	128.6±0.89	117.9±0.54	135.6±0.56	68.9±0.99
POP6	130.2±0.73	127.8±0.94	168.2±0.76	57.2±0.67
POP7	116.5±0.98	124.7±0.32	150.9±0.89	55.9±0.71
POP8	125.2±0.42	141.9±2.1	171.1±0.32	59.9±0.45
POP9	124.3±0.37	135.4±0.91	146.9±0.43	69.4±0.5
POP10	113.5±0.31	149.5±0.87	129.4±0.76	66.9±1.2
POP11	138.3±0.54*	146.2±0.98	164.1±0.67	65.4±0.98
POP12	146.9±1.01**	148.5±0.78	156.0±0.83	58.3±0.23
POP13	308.791±0.87***	244.364±0.89***	382.657±0.88***	103.847±1.2***

The values are the mean of three replications shown with standard error. One-Way ANOVA analysis were performed with repeated measures using Tukey test by GraphPad Prism 6.01v (USA).

*** showed values are p<0.0001

** showed values are p<0.001

* showed values are p<0.05

In case of GA content, a similar trend was observed. Pop13 showed significantly (p<0.001) higher amount (62%) of GA content as compared to other populations. Thus, was followed by Pop2 and Pop3 respectively (Table 3). GA content of Pop4, Pop10, Pop11, and Po12 showed almost similar amounts as compared other population, which were significantly (*p<*0.05) lower (~4.8 to 14.2%) than this. Pop1 has shown the least GA content as compared to other populations (Table 3).

The SA content was accumulated in significantly (p<0.05) higher (~three-fold) amounts in various populations specifically in Pop13 as compared to other samples. This was followed by Pop2 samples containing higher SA. Pop3, Pop8 and Pop11 showed a similar content of SA, so as by Pop4, Pop7, Pop9, and Pop12 (Table 3). The SA content in these population was statistically not significantly different (p<0.06 to p<0.59) from each other. The Kinetin levels were significantly (p<0.05) lower (20.1 to 80%) as compared to the contents of SA, GA and IAA. However, even said that, the Kinetin content was significantly (p<0.001) higher (almost two-fold) in Pop13 as compared to other twelve population of *B*. *scara* trees. The least Kinetin levels were found in Pop2 where the other had a moderate level of Kinetin as compared to Pop13 (Table 3).

### Variation of endogenous phytohromones in different population of *B*. *sacra*

**Fig 7:**
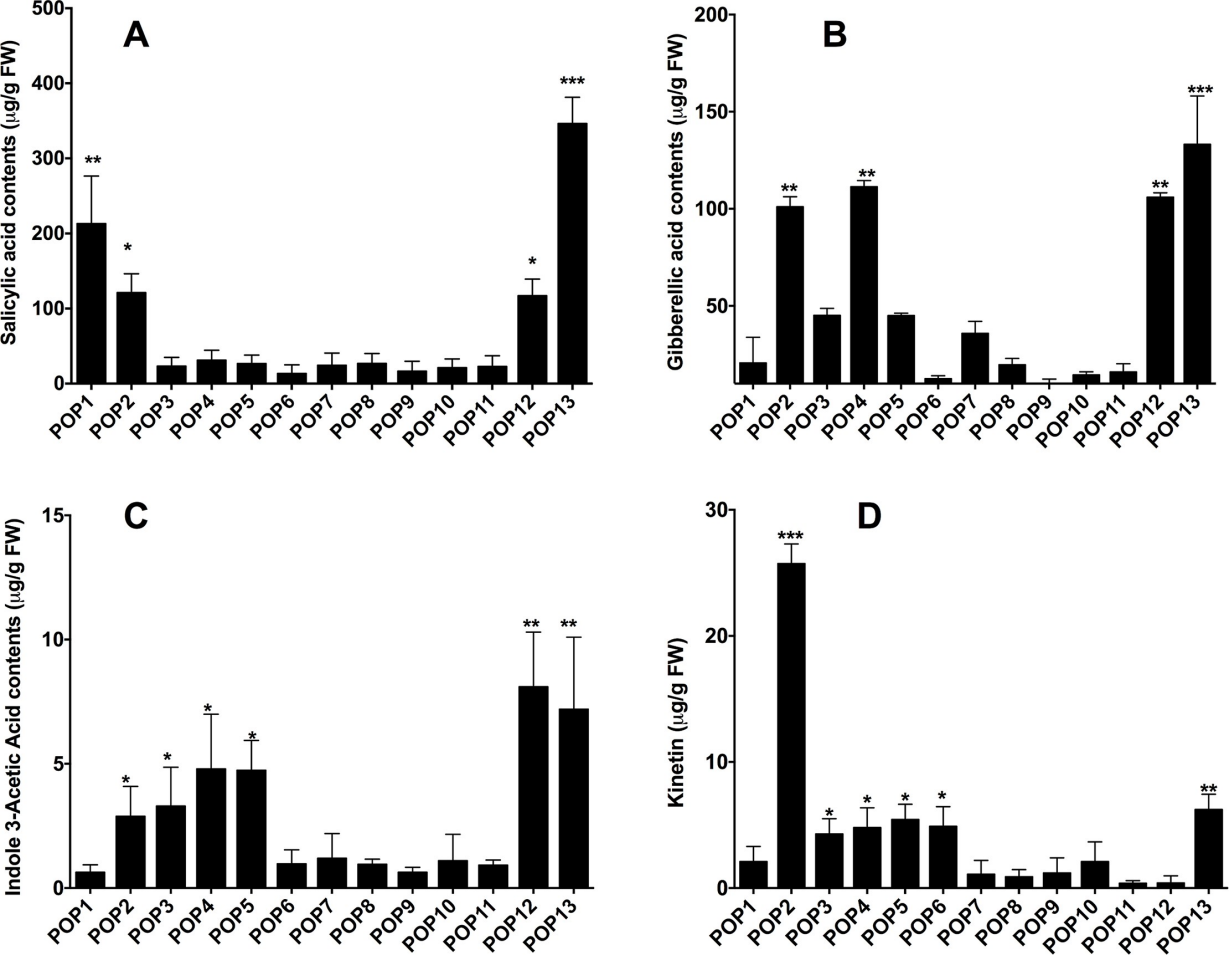
Endogenous phytohormonal quantification. Phytohormonal quantification (IAA, SA, Kinetin and GA) throught advanced chromatographic and spectroscopic techniques such as LC-ESI-QTOF and GC-MS/SIM. The values are representative of three replications with standard error. One-Way ANOVA analysis were performed with repeated measures using Tukey test by GraphPad Prism 6.01v (USA). *** showed values are p<0.0001; ** showed values are p<0.001; * showed values are p<0.05.

We have analyzed the endogenous phytohormones SA, GA, IAA and kinetin from the thirteen different populations by liquid chromatography electrospray-ionization quadrupole time-of-flight (LC-ESI-QTOF) and gas chromatograph mass spectroscopy with selected ion monitor (GC-MS/SIM). The SA levels were significantly higher (p<0.0001; ~4.8-fold) in Pop13 as compared to other populations. Besides, Pop1 and Pop2 also had significantly higher (p<0.001; ~2.4-fold) SA contents as compared other populations (Fig 7). In case of GA, it was significantly higher (p<0.0001; ~2 to 3-fold) in Pop2, Pop4, Pop12 and Pop13 as compared to other populations of *B*. *sacra* tree. The IAA content across *B*. *sacra* population also showed a similar trend like GA, however, the quantities were significantly lower. IAA content was significantly higher in Pop2, Pop4, Pop5, Pop12 and Pop13 (Fig 7). The kinetin showed a varying response compared to SA, GA and IAA as it was significantly higher only in Pop2 (Fig 7). Additional, non-metric multidimensional scaling (nMDS) analysis was performed for all samples and their phytohormonal contents. The nMDS analysis revealed that Pop13 and Pop2 were distantly apart from the rest of the population in phytohormonal contents. This nMDS analysis further supports our results from PLS model. nMDS analysis further evidence that Pop11/Pop12, Pop7/Pop9, Pop4/Pop10 and Pop1/Pop5 formed adjacent groups (S1 Fig).

## Discussion

The current study for the first time demonstrates the possible use of robust NIR based techniques to understand the tree population health and growth by quantifying the endogenous phytohormones. Specific plant community responses of endogenous phytohomones have been least studied. Phytohormonal changes in plants are cued in response to plant growth, development and abiotic or biotic stress responses[16,53]. This fluctuation could range from an individual tree to the whole population of the same tree. Such variation in the plant phytohormonal contents can also be attributed to the climatic condition, in which they are growing [36]. Increased contents of phytohormones suggest up-regulation of plant’s internal metabolism to growth or climatic conditions [54,55]. In the current study, we found that the endogenous phytohormones varied across population of the same species. The results showed that the trend in phytohormonal content increased in SA>IAA/GA>Kinetin. Among plant hormones, SA has been known as archetypal defense hormones. Increased activation of SA in Pop13 shows the enhanced modulation to disease resistance and abiotic stress mitigation. This could also be attributed to the fact that the *B*. *sacra* trees growing in the Pop13 are often exposed to salinity (on the banks of Arabian Sea) and high heat stress. Though, during the field observations we did not specifically found traces of pathogenic incidences on the tree population (Pop13) at the end of Hasik area, however, the insect (ants) were abundantly surviving in the tree species.

Plants own multifunctional and quickly advancing particular metabolites. Numerous metabolites don't seem, to be promptly required for survival; nevertheless, several may contribute to maintain population fitness in fluctuating and geologically scattered conditions [56]. Increased SA content has been argued with pathogenic attack through a combination of constitutive defense responses termed as systemic acquired resistance [57–60]. However, this could also be contributed to the abiotic stress conditions confronted to the tree population. During severe stress conditions, the SA increases which can be also attributed to the plant responses to oxidative stress [61,62]. Similarly, the Pop2, growing in the Dowkah valley, showed a distinctive endogenous phytohormonal profile with significantly higher SA levels. Though, this population is 50km away from the coastal line as compared to Pop13, but this too has been exposed to harsh environmental conditions with higher frequencies of heat stress. Some of the findings of the current study are also in conformity with the Corcuera [23] and Kroes [63] who have suggested that among factors, arid conditions for *Pinus pinaster* populations can result in varying responses of proline and phytohormone levels. Similar results of significantly altered endogenous abscisic acid (ABA) and gibberellins contents were reported in seed collected from two population of Mediterranean shrub, *Cistus albidus* L. grown in two ecologically contrasting environments in north eastern-Spain by Siles et al. [64]. Furthermore, Corcuera et al. [65] also reported the abscisic acid, (ABA), jasmonic acid (JA), indole acetic acid (IAA) and salicylic acid (SA) accumulation in two *Pinus pinaster* provenance-progeny trial sites. Moreover, Population-level quantitative variations in herbivore-elicited metabolites only partly overlaps with jasmonate accumulation polymorphisms [66]. Recently, Kroes [63] has shown that factor such as herbivore can drastically affect wild populations of cabbage whilst responding through increased phytohormonal synthesis. In addition, Dicke [57] suggested that endogenous phytohormone plays a vital role in specific plant community and ecology.

Environmental stresses represent the most limiting factors to plant growth and productivity [67]. But as the plants are sessile and cannot escape from changing environmental conditions [68], therefore they have dynamic frameworks capable to continuously adjust to changing environmental conditions, demonstrating an amazing phenotypic plasticity [69]. This is especially beneficial in heterogeneous situations to adapt to such adverse situations, plants have evolved well-developed mechanisms that help to identify the stress signals and enable optimal growth response [70]. Phytohormones mainly contribute in plants adaptation to adverse condition [67]. The intricate hormone signaling and their capability to crosstalk, make them ultimate candidate for intervening defense responses against various stresses [71]. In current study SA, gibberellic acid (GA) and IAA were considerably higher in Pop13 collected from the *B*. *scara* population limits. A similar but a little lesser content of GA and IAA were found in Pop2 (Dowkah valley) which is a starting point and the other extreme of *B*. *sacra* population. It is well reported that several strain-responsive genes respond to plants endogenous hormones, which have already been documented showing that auxin (AUX), gibberellins (GA), cytokinins (CK), abscisic acid (ABA), brassinosteroids (BRs) and salicylic acid (SA) are involved in stress signaling [72]. GA and IAA have been coined for their functional role in plant growth and development as well as in tolerance of plant during abiotic and biotic stress factors such as salinity, drought, and heat etc. [73–75]. Similarly, kinetin was significantly higher in Pop13 but least in Pop2 as compared to the other populations of *B*. *sacra*. Kinetin, on the other hand, in conjugation with IAA can help in increasing the cell division [76]. In addition, kinetin helps the plant to counteract the abiotic stress whilst maintaining a sturdy growth [77].

The eco-physiological interactions of phytohormones within the plant community have considerable implications on population dynamics, regeneration capacities and responsiveness to stress factors [78]. In addition, phytohormonal fluxes are often altered on the bases of plant’s age but such alteration does not impact leaf growth [79]. In the current study, sample from trees with uniform height, width, leaf size and age were selected to minimize such alterations. Further to this, enough biological replications and extra-ordinarily large sample size have promoted the reduction of the phytohormonal iteration across different population. Current NIRS data of phytohormonal quantification coupled with regression modeling were cross validated by advanced chromatographic and spectroscopic techniques such as LC-ESI-QTOF and GC-MS/SIM [51,80]. This was ensuring the reliability of NIRS model and to understand whether the data analyzed by both the methods show similar trends or not. The comparative assessment varied in concentrations between the two methods of analysis, however, it is worth mentioning that the data showed similar trends in phytohormonal profiling of the 13 populations studied, suggesting the authentication of the model.

In conclusion, the responses of the endogenous phytohormonal synthesis in a tree community are similar to each other but can exhibit variations, which usually depends on the growth conditions and exposure to stress. It is suggested that this newly proposed Near Infrared spectroscopy together with partial least square regression analysis is an alternative, robust, nondestructive and a rapid quantification tool for the analysis of plants phytohormones i.e. gibberellic acid, indole acetic acid, kinetin, and salicylic acid to understand their variation among 13 different populations of *B*. *sacra* tree. The results showed that the phytohormonal contents varied significantly (p<0.001) across different populations. Among phytohormones, SA content was highly significant in its content as compared to other phytohormones. Thus, the overall trend in phytohormonal content is SA>IAA/GA>Kinetin. PLS regression models made using NIRS spectral information were in total agreement with the current investigations of phytohormone analysis. The method presented herein is an alternative approach to understand forest ecology and tree health and can also help in adopting conservation strategies used for species with low regeneration issues such as *B*. *sacra*.

## Supporting information

S1 Table Details of endogenous salicylic acid analysis of *B*. *sacra* populations. (DOC)

S2 Table Details of endogenous Gibberellic acid analysis of *B*. *sacra* populations. (DOCX)

S1 Fig Nonmetric Multidimensional Scaling (NMDS) plots for Bray–Curtis distances of different populations of *B*. *sacra* and their phytohormonal contents. The nMDS plot was made in PAST v3.0 (New Zeeland).
(DOCX)
