# Supplementary material for: Endogenous phytohormones of frankincense producing Boswellia sacra tree populations
Source: PLoS One. 2018 Dec 19;13(12):e0207910. doi: 10.1371/journal.pone.0207910 (PMC6300221; doi:10.1371/journal.pone.0207910)
Supplement: S1 Table — (DOC) [file pone.0207910.s001.doc]

**S1 Table** Details of endogenous salicylic acid analysis of *B. sacra* populations

HPLC conditions used for salicylic acid analysis.

| **Gradient** | **5min** | **2.5min** | **4.5min** | **5min** | **3min** |
| --- | --- | --- | --- | --- | --- |
| Solvent A | A : 30% | A : 40% | A : 60% | A : 30% | A : 30% |
| Solvent B | B : 70% | B : 60% | B : 40% | B : 70% | B : 70% |

Solvent A = 100% MeOH; Solvent B = 100% water in 0.5% acetic acid
