# Supplementary material for: Endogenous phytohormones of frankincense producing Boswellia sacra tree populations
Source: PLoS One. 2018 Dec 19;13(12):e0207910. doi: 10.1371/journal.pone.0207910 (PMC6300221; doi:10.1371/journal.pone.0207910)
Supplement: S2 Table — (DOCX) [file pone.0207910.s002.docx]

**S2 Table** Details of endogenous Gibberellic acid analysis of *B. sacra* populations

**HPLC operation conditions for the analysis of gibberellic acid.**

| HPLC | Waters model 510 |
| --- | --- |
| Column | μ Bondapak C_18_ (3.9×300㎜) |
| Solvent A | 28% MeOH in 1% HOAc |
| Solvent B | 100% MeOH |
| Gradient | 100% Solvent A → 100% Solvent B → 100% Solvent B  (0~5 min.) (5~36 min.) (36~40 min.) |
| Flow rate | 1.5 ㎖/min. |

**GC-MS conditions used for analysis and quantification of gibberellic acid.**

| **GC-MS** | |
| --- | --- |
| Equipment | Hewlett-Packard 6890, 5973N Mass Selective Detector |
| Column | HP-1 capillary column  (30m×0.25㎜ i.d. 0.25㎛ film thickness) |
| Carrier gas | He (40 ㎖/min.) |
| Source temperature | 250℃ |
| Oven conditions | GA : 60℃(1 min.) → 15℃/min. → 200℃(1 min.) →  5℃/min. → 285℃(5 min.) |
| Injector temperature | 200℃ |
| Ionizing voltage | 70 ev |

**GC-MS SIM analysis of HPLC fractions from acidic ethyl acetate fractions of *B. sacra***

| Fraction  no. | GAs | KRI^z^ |  | *m/z* (%, relative intensity of base peak)^y^ | | | | |
| --- | --- | --- | --- | --- | --- | --- | --- | --- |
| 32, 33 | GA_4_ | 2506  2506 | sample  standard | 284(100)  286(100) | 225(80)  227(76) | 289(70)  291(71) | 224(76)  226(75) | 418(26)  420(23) |

^z)^ KRI, Kovats retention index. ^y)^ Identified as methyl ester trimethylsilyl ether

derivatives by comparison with reference spectra and KRI data (Gaskin and

MacMillan, 1991).

**Detailed method for extraction of endogenous GAs**

The extracts were passed through a Davisil C18 column (90–130 µm; Alltech, Deerﬁeld, IL, USA). The eluent was reduced to near dryness at 40 °C in vacuum. The sample was then dried onto celite and loaded onto SiO_2_ partitioning column (deactivated with 20% water) to separate the GAs as a group from more polar impurities. GAs were eluted with 80 ml of 95: 5 (v ⁄ v) ethyl acetate (EtOAc): hexane saturated with formic acid. This solution was dried at 40 °C in vacuum, re-dissolved in 4 ml of EtOAc, and partitioned three times against 4 ml of 0.1 M phosphate buffer (pH 8.0). A drop-wise addition of 2 N NaOH was maintained during the ﬁrst partitioning to neutralize residual formic acid. One-gram polyvinylpolypyrrolidone (PVPP) was added to the combined aqueous phases, and this mixture was slurried for 1 h. The pH was reduced to 2.5 with 6N HCl. The extract was partitioned three times against equal volumes of EtOAc. The combined EtOAc fraction was dried in vacuum, and the residue was dissolved in 3 ml of 100% MeOH. This solution was dried on a Savant Automatic Environmental Speedvac (AES 2000, Madrid, Spain). The dried samples were subjected to high performance liquid chromatography (HPLC) using a 3.9×300 m Bondapak C18 column (Waters Corp., Milford, MA, USA) and eluted at 1.0 ml/min with the following gradient: 0 to 5 min, isocratic 28% MeOH in 1% aqueous acetic acid; 5 to 35 min, linear gradient from 28% to 86% MeOH; 35 to 36 min, 86% to 100% MeOH; 36 to 40 min, isocratic 100% MeOH. Forty-eight fractions of 1.0 ml each were collected (Supplementary data 2). The fractions were then prepared for gas chromatography/mass spectrometry (GC/MS) with selected ion monitoring (SIM) system (6890N Network GC System, and 5973 Network Mass Selective Detector; Agilent Technologies, Palo Alto, CA, USA). For each GAs, 1 μl of sample was injected in GC/MS SIM (Supplementary data 2). Full-scan mode (the first trial) and three major ions of the supplemented [17-^2^H_2_] GAs internal standards and the plant’s GAs were monitored simultaneously whereas the same was done for endogenous GAs of both kinds of tree samples. The endogenous plant’s GAs were calculated from the peak area ratios of sample GAs to corresponding internal standards as suggested by Lee et al. (1998). The retention time was determined using hydrocarbon standards to calculate the KRI (Kovats retention index) value. The GA quantification was based on the peak area ratios of non-deuterated (extracted) Gas to deuterated GAs (Supplementary information 2; Khan et al., 2012).

**References**

Khan, A.L., Hamayun, M., Kang, S.M., Kim, Y.H., Jung, H.Y., Lee, J.H. and Lee, I.J., 2012. Endophytic fungal association via gibberellins and indole acetic acid can improve plant growth under abiotic stress: an example of Paecilomyces formosus LHL10. *BMC microbiology*, *12*(1), p.3

Lee, I.J., Foster, K.R. and Morgan, P.W., 1998. Photoperiod control of gibberellin levels and flowering in sorghum. *Plant physiology*, *116*(3), pp.1003-1011.
