# Supplementary material for: Endogenous phytohormones of frankincense producing Boswellia sacra tree populations
Source: PLoS One. 2018 Dec 19;13(12):e0207910. doi: 10.1371/journal.pone.0207910 (PMC6300221; doi:10.1371/journal.pone.0207910)
Supplement: S1 Fig — The nMDS plot was made in PAST v3.0 (New Zeeland). (DOCX) [file pone.0207910.s003.docx]

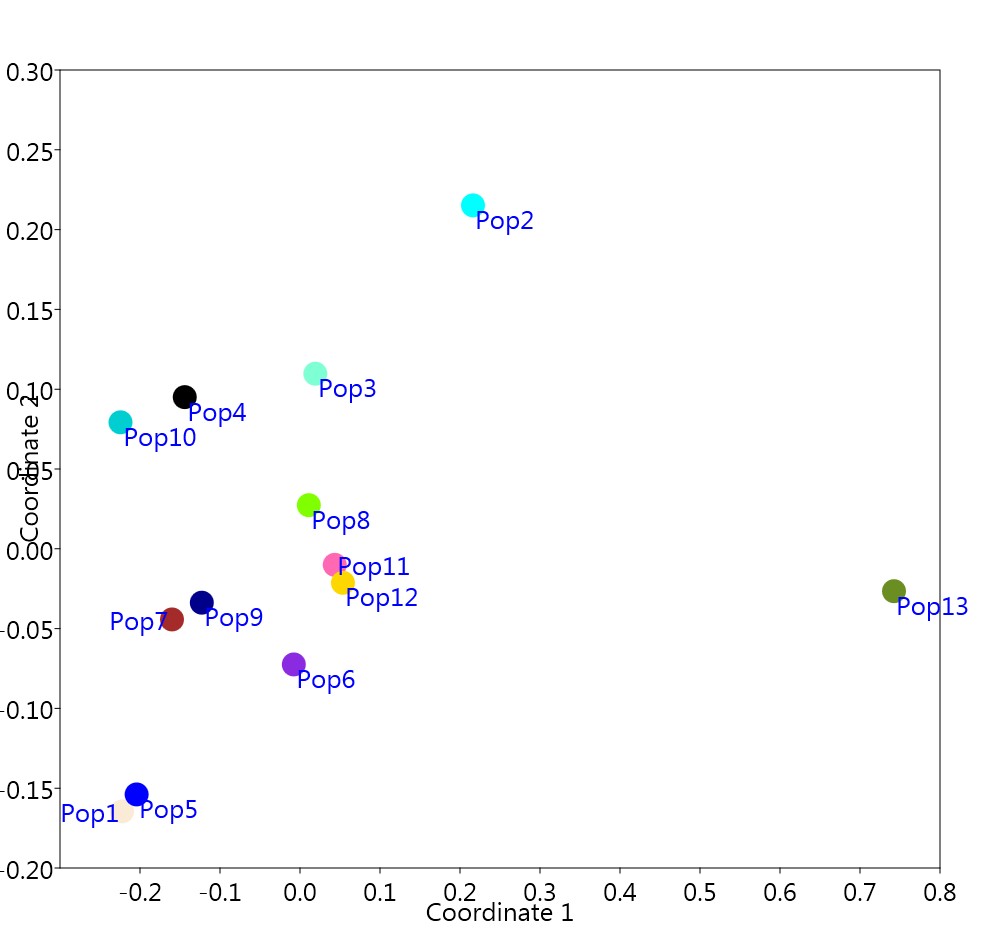


**S1 Fig.** Nonmetric Multidimensional Scaling (NMDS) plots for Bray–Curtis distances of different populations of *B. sacra* and their phytohormonal contents. The nMDS plot was made in PAST v3.0 (New Zeeland).
